# The implication gap: Why evidence-based dental guidelines fall short in clinical settings

**DOI:** 10.34172/joddd.025.44440

**Published:** 2025-12-31

**Authors:** Naser Asl Aminabadi, Katayoun Katebi

**Affiliations:** ^1^Department of Pediatric Dentistry, Faculty of Dentistry, Tabriz University of Medical Sciences, Tabriz, Iran; ^2^Department of Oral and Maxillofacial Medicine, Tabriz University of Medical Sciences, Tabriz, Iran

## Introduction

 The implementation of evidence-based dental practice guidelines is widely recognized as a cornerstone for improving patient outcomes, standardizing care, and reducing unnecessary variations in clinical practice. Guidelines covering areas such as caries management, periodontal therapy, antibiotic prescription, radiographic assessment, and infection control are readily available and are often based on rigorous systematic reviews. Despite this wealth of information, adherence in real-world dental clinics remains inconsistent and, in some cases, suboptimal.^1^ This discrepancy is often mistakenly attributed to a lack of motivation, awareness, or commitment among clinicians. A closer examination, however, reveals that systemic, structural, cognitive, and patient-related factors play a far more significant role in limiting guideline adoption.^2^

## Economic Mismatch

 One of the primary obstacles is the economic mismatch inherent in guideline implementation. Many recommendations assume ideal conditions, including sufficient appointment times, auxiliary staff support, and access to advanced diagnostic tools or restorative materials. In reality, dental clinics often operate under financial constraints driven by productivity. Time-intensive preventive interventions, patient education, or additional diagnostic procedures may not be reimbursed or may reduce the overall number of patients seen per day. As a result, clinicians are often forced to balance the ideal standard of care with financial sustainability, prioritizing efficiency over strict adherence to guidelines.^3^ For example, the use of caries risk assessment tools or preventive sealants, though strongly recommended in pediatric and adult preventive care guidelines, may be omitted in high-volume practices where reimbursement is limited, not because of clinical negligence, but due to economic necessity.^4^

## Complexity of Guidelines

 Another barrier is the guidelines’ usability and complexity. Guidelines are typically designed for academic or professional audiences and are often dense, lengthy, and written in highly technical language. While this thoroughness ensures scientific rigor, it can render the guidelines impractical for a busy clinician facing a full patient schedule. The lack of simplified decision trees, concise checklists, or stepwise algorithms makes on-the-spot application difficult. Consider infection-control guidelines: while detailed recommendations on sterilization, personal protective equipment, and procedural protocols exist, busy practitioners may struggle to consistently integrate all recommendations without streamlined tools, reminders, or electronic prompts embedded in their workflow.^5^ This usability gap forces clinicians to interpret or selectively apply recommendations, introducing variability in care and reducing guideline fidelity.

## Patient Variability

 Real-world patient variability further complicates adherence to guidelines. Most guidelines are developed based on controlled clinical trials or consensus panels and assume “ideal” patient conditions. In practice, patients present with complex combinations of medical comorbidities, behavioral factors, socioeconomic constraints, cultural diversity, and varying degrees of treatment compliance. For instance, periodontal treatment protocols may recommend multiple follow-up visits and adjunctive therapies; however, patients with limited transportation options, financial barriers, or competing health priorities may not be able to attend these appointments consistently.^6^ Clinicians, therefore, must adapt protocols to accommodate patient realities, which may appear as deviations from the guidelines. These necessary adaptations highlight the challenge of translating standardized recommendations into flexible, patient-centered care.

## Cognitive Overload

 A fourth, often overlooked factor is cognitive overload and guideline fatigue. The modern dental professional is expected to assimilate an ever-growing body of recommendations, spanning infection control, caries management, radiographic protocols, ergonomics, drug prescription, antibiotic stewardship, and preventive strategies, among others. The accumulation of these guidelines can overwhelm cognitive capacity, leading to selective attention, prioritization based on perceived relevance, or inadvertent omission of certain recommendations. Cognitive science research suggests that excessive information can impair decision-making and compliance, a phenomenon sometimes described as decision fatigue.^7^ Over time, the cumulative burden of multiple guidelines contributes to inconsistent adherence, independent of a clinician’s motivation or knowledge.

 Addressing these challenges requires a systemic, multi-faceted approach. Simplifying guideline presentation through structured summaries, flowcharts, and digital decision-support tools can reduce cognitive load and improve usability. Integrating guideline prompts into electronic dental records or practice management software ensures that clinicians receive actionable information at the point of care. Aligning reimbursement models with evidence-based practices can mitigate economic barriers, incentivizing clinicians to adopt recommended protocols without financial penalty. Incorporating patient variability into guideline development and offering flexible implementation pathways can bridge the gap between controlled research conditions and real-world practice, allowing for tailored, patient-centered care. Finally, embedding continuous professional development and reinforcement mechanisms into daily workflows can counteract fatigue and sustain adherence to guidelines over time.

 Interventions should extend beyond dissemination and education to address structural and systemic barriers. Economic incentives, workflow redesign, supportive technology, and culturally sensitive patient engagement strategies are all necessary to translate guidelines into consistent clinical practice. Implementing feedback systems to gather insights from clinicians regarding the challenges they face with guidelines can improve future revisions and ensure that guidelines are practical and relevant to everyday practice. Only by acknowledging the full complexity of real-world practice can evidence-based recommendations fulfill their intended purpose of improving oral health outcomes.

## Conclusion

 In conclusion, the gap between the publication of dental guidelines and their clinical adoption is not primarily a reflection of clinician negligence or lack of awareness. Instead, it results from structural, cognitive, and patient-centered challenges that limit the feasibility of guideline implementation. Economic constraints, usability issues, patient variability, and guideline fatigue collectively undermine adherence in daily practice. Recognizing and addressing these factors is essential for transforming evidence into actionable, sustainable care. By shifting the focus from individual compliance to system-level support, the dental profession can move closer to achieving the ideal of evidence-informed, patient-centered care, ultimately improving oral health outcomes at the population level.

## Competing Interests

 The authors are the Editor-in-Chief and Assistant Editors of the Journal of Dental Research, Dental Clinics, Dental Prospects (JODDD). The authors declare no other competing interests concerning authorship and/or publication of this article.

## Ethical Approval

 Not applicable.
